# Supplementary material for: Are carbon emissions trading and green financial instruments synergistic? -Comprehensive quantitative research based on content analysis
Source: PLoS One. 2024 Mar 7;19(3):e0298601. doi: 10.1371/journal.pone.0298601 (PMC10919601; doi:10.1371/journal.pone.0298601)
Supplement: S1 Data — (ZIP) [file pone.0298601.s001.zip › Data/地级市Z_F工作报告.zip!/╕ú╜¿/─■╡┬╩╨/└┤╫╘╣1⁄2╓┌║┼í╛┬φ┐╦╩2╛▌═°í┐_2006.docx]

各位代表：

现在，我代表宁德市人民政府向大会作政府工作报告，请予审议，并请市政协各位委员和其他列席人员提出意见。

一、回首“十五”，全市经济社会发展取得重大成就

2005年，在省委、省政府和市委的正确领导下，我市各级政府以开展保持共产党员先进性教育活动为动力，认真贯彻党中央、国务院和省委、省政府一系列决策部署，全面落实科学发展观，努力构建社会主义和谐社会，攻坚克难，致力发展，经过全市人民共同努力，较好完成了市一届人大七次会议确定的经济和社会发展各项目标任务。

国民经济持续快速增长，综合实力进一步提升。全年生产总值342.3亿元，增长10.6%。财政总收入（不含基金收入）20.3亿元，可比增长29.2%，其中地方财政收入12.65亿元，可比增长25.2%。农业生产和农村经济在大灾之年继续保持稳定增长，全年完成农林牧渔业总产值143.4亿元，增长4.2%。农业产业化步伐加快，全市市级以上农业龙头企业增加到88家，有22个农产品获省名牌产品或著名商标称号， 8个品牌被授予2005年中国“三绿工程”推荐品牌，6个产品获得地理标志证明商标，居全省第二位。工业经济保持快速增长，全年完成工业总产值294.7亿元，比增22.4%；其中规模以上工业产值187.1亿元，比增33.8%，增幅居全省第一。完成工业技改投资24亿元，增长30.4%。以旅游为重点的第三产业进一步发展。太姥山、白水洋被列入国家地质公园，全市共接待游客345万人次，实现旅游总收入24亿元，分别增长15%和20%。金融保险、交通运输、房地产、信息和中介服务等服务业加快发展。全年实现社会消费品零售总额123.8亿元，增长11.8%，居民消费价格指数102.4%。

项目带动力度加大，发展动力进一步增强。全年完成全社会固定资产投资135.5亿元，增长33.1%。实施重点建设项目46个，完成投资53.7亿元，占全社会固定资产投资的39.6%，增长34%。共有7个项目实现竣工投产或部分投产，18个项目完成或超额完成年度计划，其余项目也都按计划扎实推进。重点建设项目中工业及能源项目22个，完成投资41.6亿元，占重点建设项目完成投资的77.5%，进一步增强了工业经济发展后劲。中心城市城建项目全年完成投资5.88亿元，有42个项目实现年度建设目标。

改革开放继续深化，发展活力进一步激发。继续深化国有企业改革，健全完善国有资产管理体制。闽东电力股份公司扭转危机、治理整顿取得成效，全面实现扭亏增盈，实现扭转ST的预期目标。市外贸企业总体完成改制，市属内贸企业改革已经启动。加大对民营经济的政策支持，非国有工业产值占全市工业总产值的比重提高到96.8%。稳步推进农村各项改革，继续实行最低收购价、种粮直补、良种补贴，全部免征农业税，取消烟叶外的农业特产税，基本完成集体林权制度改革任务。成立了省农村信用联社宁德办事处。积极组织境内外、省内外各种招商活动，举办形式多样的招商节会，全年实际利用外商直接投资1.14亿美元（报表口径），增长26.1%；外贸出口总额3.25亿美元，增长26%。城澳口岸通过国家级一类口岸验收，并经国务院批准更名扩大为宁德港口岸对外开放。

社会事业协调发展，统筹能力进一步提高。科技工作取得实效，有3个产品获得福建省优秀新产品，2个新产品通过省级新产品鉴定，3个项目被列为省循环经济百项重点示范项目，福建鑫磊晶体有限公司被列为省循环经济百家重点示范企业。认真落实全省教育振兴行动计划实施意见，加大对教育的资金投入和政策倾斜，中小学危房改造步伐加快，宁德市职业技术学院正式获准成立，宁德师专新校区征地工作基本完成。卫生工作得到加强，全面实施乡镇卫生院危房改造和“空白点”村卫生所建设，扩建市医院传染病房，建成市疾病预防控制中心综合楼、市康复医院病房主体工程。加强文化基础设施建设，完成市文化中心主体工程，畲族文化抢救保护工作得到加强。群众性体育运动广泛开展，我市运动员在第十届全运会上取得三金一银一铜的历史最好成绩。其它各项事业同步协调发展，去年我市再次荣获全省“双拥模范城”称号，第四次被国务院评为“民族团结进步模范集体”。

惠民政策较好落实，人民生活进一步改善。扎实做好就业再就业工作，全年城镇新增就业3.19万人，有7419名下岗失业人员实现再就业，城镇登记失业率4.3%；新增农村劳动力转移6.2万人次。社会保障覆盖面进一步扩大，全市城乡共有13.37万人享受低保，城镇职工养老、失业、医疗保险参保人数分别达14.7万人、10.7万人、15.03万人。有效抵御连续频发的台风、洪涝等自然灾害，扎实推进灾后恢复生产和重建家园工作，因灾倒房的2413户春节前都搬进新居。认真实施老区工作优先行动，深入开展扶贫攻坚。全年完成“造福工程”和滑坡险情村搬迁8822人。实行农村低保家庭子女义务教育“两免一补”制度。认真清理拖欠工程款和农民工工资问题。全市共偿还政府投资项目拖欠工程款1.1亿元，清欠率97.6%。开展新型农村合作医疗试点。做好高致病性禽流感等重大传染病的防控工作。进一步强化食品药品安全质量监管。城乡居民生活水平继续提高。全年城镇居民人均可支配收入9785元，增长9.8%；农民人均纯收入3808元，增长8.3%。

社会保持团结稳定，发展合力进一步形成。自觉接受人大及其常委会的依法监督、人民政协的民主监督和社会各方面的监督。全年共办理市人大代表建议170件、市政协委员提案167件。虚心听取各民主党派、工商联和无党派人士的意见和建议，加强了与工会、共青团、妇联等人民团体的联系。深化推进“平安宁德”建设，顺利通过全省“四五”普法验收。切实加强社会治安综合治理，严厉打击各种违法犯罪活动，继续保持刑事发案总量低于全省平均水平、打击整体效能高于全省平均水平的良好态势。认真落实安全生产责任制，安全生产形势稳定好转。高度重视群众信访工作，一批涉法涉诉、重点信访和老信访问题得到依法妥善处理。深入开展精神文明创建活动，涌现出一批国家级、省级文明单位典型。国防动员、民兵预备役、海防、人防等工作继续加强。认真落实国务院依法行政实施纲要，深入开展绩效评估和“行政提速、服务发展”活动，进一步优化了政务环境。扎实推进政务公开，廉政建设和反腐败工作取得新进展。

各位代表，2005年工作任务的完成，标志着我市“十五”计划的全面超额完成，经济社会发展又迈上了一个新台阶，使宁德进入了一个蓄势待发、加快发展的新阶段，也为“十一五”发展奠定了良好基础。

“十五”时期，是经济总量快速扩张的五年。五年间，全市GDP年均增长10%，高于“十五”计划1个百分点；2005年末人均GDP达11222元，超过“十五”计划目标，年均增长9.6%。财政总收入和地方财政收入同口径都比“九五”末翻了一番，分别年均增长15.5%和16%，高于“十五”计划目标5.5和6个百分点。市本级和柘荣县连续五年实现当年财政收支平衡，福鼎、福安、古田连续四年实现当年财政收支平衡。全市金融存、贷款余额双双突破200亿元，分别是“九五”末的2.4倍和2.5倍，也实现了翻番。

“十五”时期，是工业化进程加速推进的五年。五年间，全市工业总产值年均增长16.8%，其中规模以上工业产值年均增长28.6%，2005年规模工业总量比“九五”末翻了近两番，工业化水平由“九五”末的24%提高到28%。初步形成了电机电器、船舶修造、医药化工、食品加工、汽摩配件、建筑建材、能源等七个重点、特色产业，其中电机电器、石材被列入省第一批重点培育建设的产业集群。全市现有超亿元企业45家，7家行业龙头企业被确定为福建省百家重点企业，工业系统拥有省“名牌产品”24个，省“著名商标”11个。福建三祥冶金被科技部授予高新技术企业称号，安波电机获国家免检产品称号，实现历史性突破。全市已开发工业集中区27个，落户企业860家。积极谋划和争取一批临海重化产业项目。宁德大唐火电一期120万千瓦即将投产，实现了我市在布局大项目上零的突破。大唐火电二期、宁德核电厂一期项目前期工作顺利推进，大型钢铁、石化、港口物流等项目前期工作不断深化，签约并启动了一批如第六代集装箱码头、城澳30万吨油品码头、安发国际益槐（福建）生物科技园等项目。

“十五”时期，是城乡基础设施建设取得重大突破的五年。全市累计完成全社会固定资产投资449.5亿元，超额完成“十五”计划149.5亿元，是“九五”时期的2.5倍。建成了一大批交通、能源等基础设施项目，是宁德历史上投资项目最多、投资额最大、经济社会效益最好的时期。福宁高速公路及福安连接线建成通车，城澳万吨级码头顺利竣工，温福铁路宁德段全面动工。市县交通网建设投入7.35亿元，完成农村道路硬化1780公里。建成投产穆阳溪一级芹山、二级周宁水电站、寿宁牛头山水电站、屏南上培、万坑水电站、古田双口渡水电站，完成建设改造22万伏宁德兰田变电、福鼎桐城变电以及农村和县城电网建设，洪口电站、穆阳溪三级闽东电站、宁德50万伏开关站以及宁德电力输配电网络改造与建设工程等一批电源点和电网项目正在加紧建设。累计投入7.12亿元，改造了2173个行政村电网，受益农户40多万户200多万人。防灾减灾体系建设得到加强。全面完成造林任务和沿海防护林建设，完成了三沙供水工程建设，赛江下游防洪一期工程、宁德市防洪防潮工程等一批水利重点项目全面启动。水利“六千”工程建设扎实推进，建成农村饮水工程502个。同时，一批重大出省交通通道前期工作实现突破，宁武高速公路预可通过国家评估，宁衢铁路前期工作取得重要进展，古田高速公路连接线、福安至泰顺高速公路、同三线福鼎段高速连接浙江甬台温高速复线前期工作正在加快推进。

“十五”时期，是城市化水平明显提升的五年。确定了宁德中心城区东扩、南移、面海和向海、临海、跨海的发展布局并在加紧建设中，城市用地和人口规模已获国家批准。投入了中心城市政府性市政建设资金11.5亿元，建成了一大批城市基础设施工程和社会事业项目，城市 “四纵四横” 路网框架基本形成，东侨新区初具规模，金港围垦、铁基湾围垦正在加快实施。市区总人口增加到18万人，比 “九五”末增加7.6万人；建成区面积扩展到11.8平方公里，比“九五”末扩大4平方公里。全市城镇化步伐明显加快，各县（市、区）五年累计投入政府性城市市政设施建设资金18.1亿元，建成区面积达到51.6平方公里，比“九五”末增加11.85平方公里。全市累计完成房地产开发投资64.3亿元，是“九五”时期的4.9倍，商品房竣工面积288.5万平方米，城镇居民人均住房面积34.6平方米,比“九五”末增加13.1平方米。2005年末，全市城镇化水平38.2%，高于“十五”计划2.2个百分点。

“十五”时期，是改革开放和区域合作纵深推进的五年。各项改革稳步推进。基本完成国有工业企业改革任务。重组了市国投公司、城投公司和土地收储中心。投融资、财税、流通、电力、社会事业、城市公共事业等体制改革加快推进。企业融资体系建设步伐加快，全市现已组建12家中小企业融资担保公司，累计为500多家企业融资40多亿元。制定出台促进县域经济发展的一系列措施，有力推动了县域经济发展。农村税费改革、集体林权制度改革及水利产权制度、水利管理体制改革等农村综合改革取得实效。深化政府机构和行政审批制度改革，开展“创建效能型、服务型、责任型政府，营造优良政务环境”和“行政提速、服务发展”活动，政府行政效能进一步提高。对外开放水平不断提升。积极开展招商引资工作，组建了市招商中心，每年都举办一系列招商活动，加强对外宣传推介和常年招商工作。重点强化国内外重化产业的承接和与浙南及“长三角”地区的协作对接，浙南已有200多家企业、100多亿元资金投资宁德。五年累计实际利用外商直接投资4.08亿美元， 累计出口总值10.4亿美元，分别年均增长15. 5%和34.5%，高于“十五”计划10.5和24.5个百分点。加强宁台港澳侨交流合作。开辟城澳至马祖石料运输直航航线，成功运营近百个航次，为争取宁德港成为对台固定直航点奠定了基础。

“十五”时期，是各项社会事业加快发展的五年。科技创新成效明显，颁布实施《宁德市科学技术奖励规定》，五年共实施国家、省、市各类重点科技项目261项，全市现有省级高新技术企业8家，省级企业技术中心4家。教育“两基”成果得到巩固，办学条件不断改善，“双高普九”持续推进。全市五年改造、新建中小学校舍面积45.1万平方米，新增普通高中学校11所。去年高中阶段毛入学率60.7%，高考录取率74.5%，分别比2000年提高了36个百分点和26个百分点;中等职业学校在校生达3.65万人，比2000年增长50%。高等教育有新发展，宁德师专新建扩容筹建学院和创办宁德职业技术学院正在加紧实施。人才引进力度加大，人才流失问题逐步得以控制。城乡公共卫生体系进一步完善，疾病预防控制和医疗救治体系基本建成，突发公共卫生事件预警和应急救治机制初步形成，乡镇卫生院危房改造和“空白点”村卫生所建设扎实推进。成功举办两届市运动会，推动群众性体育运动蓬勃发展。人口、资源、环境工作不断加强。古田、福鼎、蕉城先后获得省级计生优质服务先进县（市、区）称号，全市有六个县（市、区）进入省计生“三为主”二类县（市、区）行列。连续十一年实现省里下达的人口控制目标。连续七年实现耕地占补平衡。重点流域、重点区域的环境保护得到加强，城乡绿化和绿色通道建设、“青山挂白” 及石材业、养殖业污染等专项整治工作取得初步成效,环境质量继续保持良好。社会主义民主政治和精神文明建设进一步加强，民族、宗教、侨务工作和文化、新闻出版、广播电视等各项社会事业都取得新成绩。

“十五”时期，是人民生活和社会保障水平不断提高的五年。“十五”期间，全市城镇居民人均可支配收入和农民人均纯收入分别以年均10.5%和6%的速度增长。2005年城镇居民和农民恩格尔系数分别比2000年下降了2.5个百分点和3.7个百分点。实施的“16833”就业工程全面完成，累计新增城镇就业12.1万人，实现下岗失业人员再就业2.8万人。至去年底，全市已累计转移农村劳动力45万人次。社会保险、社会救助、社会福利、优抚安置、社会互助相结合的社会保障体系初步形成。老区“五通”工作取得明显成效。扶贫工作扎实推进，五年共落实省、市扶贫开发重点村扶贫项目989个，下拨捆绑资金 4200多万元。加大力度解决安居问题，累计完成“造福工程”和滑坡险情村搬迁近5万人。东侨华侨农场职工安置、就业、社保和子女入学等问题得到较好保障，受到国务院侨办、中国侨联的充分肯定。

各位代表，五年的发展成就令人鼓舞，五年的实践也使我们积累了许多宝贵的经验。这就是：必须始终坚持以科学发展观为指导，努力实现经济社会又快又好发展；必须始终坚持把发展作为第一要务，不断提升综合经济竞争力；必须始终坚持改革开放，以改革增活力，以开放促发展；必须始终坚持统筹兼顾，更加注重城乡、区域、经济社会协调发展，加快构建社会主义和谐社会；必须始终坚持依法行政、执政为民，使发展的成果惠及广大人民群众；必须始终坚持勤政廉政、从严治政，努力为发展创造优良的政务环境。

过去的五年，是宁德历史上综合实力提高最快、城市面貌变化最大、人民群众得到实惠最多的时期。这是省委、省政府和市委正确领导的结果，是全市干部群众抢抓机遇，在竞争中迎接挑战，在转型中经受考验，在前进中赢得发展的结果，也是历届政府打下基础，本届政府致力发展、攻坚克难、努力工作的结果。在此，我谨代表宁德市人民政府，向为我市社会主义现代化建设事业付出辛勤劳动的全市人民，向给予政府工作大力支持与有效监督的人大代表、政协委员和各民主党派、工商联、群众团体、无党派人士，向为宁德改革发展稳定作出积极贡献的中央及省属驻宁单位、驻宁部队、武警官兵、民兵预备役官兵、公安政法干警，向关心和支持宁德建设与发展的港澳台同胞、海外侨胞、国际友人和各界人士，表示衷心的感谢和崇高的敬意！

在充分肯定成绩的同时，我们也要清醒看到存在的困难和问题：一是经济总量小，工业化水平低，财政困难状况未得到根本好转。二是区域、城乡发展不平衡，市级经济实力较弱，对县域经济的聚集、辐射和带动能力不足。三是解决“三农”问题的任务依然艰巨，就业再就业压力比较大，部分群众生活还比较困难。四是社会事业发展相对滞后，人才缺乏，自主创新能力不强。五是安全生产基础薄弱，突发公共事件和重大自然灾害时有发生，事关群众安全健康的保障、预防、应急处置体系还有待健全完善。六是制约经济发展的体制机制矛盾尚未根本消除，消极腐败现象在一些地方和部门依然存在，一些干部的观念、作风还不完全适应形势发展的要求，发展的环境有待进一步改善。这些问题，都要在今后工作中针对性地采取有力措施加以解决。

“十一五”是宁德建设海峡西岸经济区东北翼中心城市的关键时期。去年底市委召开一届十二次全会，审议通过了《关于制定宁德市国民经济和社会发展第十一个五年规划的建议》，确立了我市“十一五”时期发展的指导思想、基本原则、目标定位和主要任务。根据《建议》市政府及时研究编制了《宁德市国民经济和社会发展第十一个五年规划纲要（草案）》，致力于把宁德建设成海峡西岸经济区东北翼中心城市、对接长江三角洲的前沿区域、对台交流合作的重要平台、联接内陆与海外的重要港口、东北翼新的增长极。主要发展目标是：在优化结构、提高效益和降低消耗的基础上，“十一五”期间全市地区生产总值年平均增长12%，到2007年地区生产总值与全省同步比2000年翻一番，2008年人均地区生产总值与全省同步比2000年翻一番，2010年地区生产总值600亿元以上，人均地区生产总值近2万元。围绕实现这些目标，《纲要》提出了“十一五”期间我市经济社会发展九个方面的主要任务和措施。根据议程安排，这次会议还将对《纲要》及《纲要》的说明一并提交大会审议。

二、把握开局，扎实做好2006年各项工作

2006年是全面实施“十一五”规划的第一年。起步关乎胜负，开局影响全局。做好今年的工作，对顺利完成今后五年的各项目标任务十分关键。今年政府工作的总体要求是：坚持以邓小平理论、“三个代表”重要思想和胡锦涛总书记视察福建的重要讲话为指导，认真贯彻党的十六大、十六届五中全会和中央经济工作会议及省委七届十次全会、全省经济工作会议精神，以科学发展观统领经济社会发展全局，全面落实市委一届十二次全会和全市经济工作会议部署，坚持以经济建设为中心，强化项目带动，突出工业主导，提高自主创新能力，转变经济增长方式，提升区域经济实力，加快社会主义新农村建设，推进和谐社会建设，提高人民群众生活水平，努力实现宁德经济社会持续快速健康发展，为 “十一五”规划的实施开好局、起好步，全面推进海峡西岸经济区东北翼中心城市建设。经济社会发展的主要预期目标是：地区生产总值增长11%；全社会固定资产投资增长12%；社会消费品零售总额增长13%；外贸出口增长18%，实际利用外商直接投资增长10%以上；地方财政收入增长12%；城镇居民人均可支配收入增长9.5%;农民人均纯收入增长6%以上；居民消费价格总水平涨幅控制在3%左右；城镇登记失业率控制在4.5%左右；人口自然增长率控制在7‰以内。

实现上述目标，必须扎实做好以下六个方面工作：

1、坚持工农与城乡统筹发展，促进社会主义新农村建设

认真贯彻“工业反哺农业、城市支持农村”和“多予少取放活”的方针，按照因地制宜、分类指导、量力而行、尽力而为的原则，尊重农民意愿，维护农民利益，增进农民福祉，扎实推进社会主义新农村建设。抓紧制定全市新农村建设总体规划，今年安排各种类型试点村100个，其中市级10个，县级90个，确保取得实实在在的进展。

坚持以发展农村经济为中心，千方百计促进农民增收。加大农业结构调整力度，保护和提高农业综合生产能力。继续认真落实扶持粮食生产的有关政策，全年粮播面积稳定在266万亩，粮食产量稳定在82万吨。大力发展高效优质生态农业，进一步做大茶叶、食用菌、水产、林业、畜牧、药材、果蔬等特色产业，搞好品牌整合和农业标准化建设，推动农业机械化发展，加快建设一批优势农业产业链、高优农业示范区和知名农产品品牌。推进农业产业化，继续实施“强龙”工程，重点扶持一批带动能力强、发展潜力大的农业龙头企业，争取全市年销售额5000万元以上的农业龙头企业增加到30家以上，销售额亿元以上的农业龙头企业达到4家，力争规模以上农业龙头企业产值增长15%以上。积极发展农村合作经济组织，发展订单农业，完善利益联结机制，不断提高农民进入市场的组织化程度。加快农村劳动力转移，继续实施农村劳动力转移培训和“一户一工”工程，力争年内新转移农村劳动力5万人以上。认真贯彻国务院《关于解决农民工问题的若干意见》，切实保障农民工合法权益。

加大对“三农”的投入力度，改善农村生产生活条件。围绕农民最关心、最直接受益的实际问题，推进新农村建设五件实事工程：一是基础设施工程。组织编制村镇布局规划，逐步把公路延伸到规划的农村居民点。抓好农村道路硬化，确保今年完成800公里。加快在建水利工程建设，确保一批重点水利水毁集中整治工程和病险水库、海堤除险加固工程今年汛前竣工。继续实施水利“六千”工程，确保完成省下达的年度建设任务。加强森林防火，分年度扎实推进1000公里生物防火林带建设。二是教育培训工程。今年起实行农村九年义务教育学杂费全免。继续实行农村低保家庭子女接受义务教育“两免一补”制度，完善贫困学生助学体系。加快整合各类培训资源，广泛开展农民职业技能培训，增强农民就业能力。三是农民健康工程。继续扩大新型农村合作医疗试点面，今年增加古田、福鼎、屏南三个试点县（市），到2008年全市全面铺开。加强农村药品监督和供应网络建设，开展农村医疗救助试点工作，落实好乡村医生补贴培训和乡镇卫生院人员工资按比例由财政拨补的政策，完善“空白点”村卫生所建设，年内完成全市33个乡镇卫生院危房改造任务。抓好高致病性禽流感等重大动物疫病的防控工作。四是环境整治工程。深入开展以“三清六改”为主要内容的村容村貌整治，今年重点抓好20个示范村的整治工作。实施农村“家园清洁行动”，今年重点治理10个乡镇、100个村，在疏浚农村河道、农村卫生改厕、污水垃圾处理和推广户用沼气等方面扩大试点，取得经验后逐步推开，切实改善农村环境面貌。五是文化建设工程。加快推进广播电视“村村通”工程，市财政从今年起，每年安排专项资金50万元，力争到2008年实现“村村通”目标。重点扶持建设一批乡镇综合性文化站，积极开展送科普、送电影、送戏下乡活动，促进先进文化在农村的传播覆盖。

深化农村综合改革，进一步增强农村发展活力。稳步推进以乡镇机构、农村义务教育、县乡财政体制改革为主要内容的农村综合改革，增加各级财政对农业的投入，认真落实各项支农惠农政策，保证已经实行的支农措施不变，已经给予农民的实惠不减。扎实抓好农业普查工作。继续深化林权、海域权制度改革。积极探索征地制度改革，稳妥推进土地使用权流转。加快农村信用社改革。进一步创新农村社会化服务体系建设，增强服务“三农”能力。

2、坚持集聚与提升有机结合，促进工业做大做强

加快产业聚集，做大工业总量。一方面，依托港口优势，临港聚集一批。按照临海产业布局规划，积极承接国内外大型重化产业转移。在确保大唐火电一期并网发电的基础上，争取上马火电二期工程，争取年内国务院核准宁德核电项目建设。继续加强与国内外大型钢铁和石化投资企业的合作，力争在引进大型钢铁、石化项目上取得新突破。加快三都澳20万吨级国际集装箱码头、30万吨级油品码头、30万吨级船坞、福宁船舶重工、福安50万吨冷轧硅钢片等一批大中项目的前期工作，促其尽快开工建设。另一方面，依托区位优势，就近聚集一批。重点加强与浙南及“长三角”的经济协作和产业对接，通过政府搭台、企业运作，着力招引一批产业转移型、资源利用型、加工贸易型和集群配套型项目。同时，大力实施一批工业生产性项目，重点抓好85个千万元以上的新增长点项目，加强协调调度，切实解决项目实施过程中的问题，促其早日投产发挥效益。

提升发展水平，做强传统产业。以提升现有传统产业为重点，实施技术创新、集群培育和品牌建设三大战略。把自主创新作为提升传统产业的中心环节，加大对电机电器、医药化工、食品加工、船舶修造、汽摩配件、建筑建材等传统产业的扶持力度，促进传统产业改造升级。把培育大企业大集团作为提升传统产业的重要举措，以骨干企业和名牌产品为核心，引导企业开展多种形式的并购重组，推进横向联合，集聚优质资产，壮大经营规模。今后，市里每两年将在全市范围内确定30家重点企业，在企业技改、创优、创牌、融资、用电、用地等方面给予倾斜扶持。对在我市组建规范化股份有限公司或被列入国家证监会上市辅导期的企业，给予奖励和重点支持。把争创品牌作为提升传统产业的有效途径，整合重点产业优势，集中打造几个公共品牌。首期先对“中国电机电器城”、“闽东大黄鱼”、“绿雪芽”茶、“柘荣太子参”、“古田食用菌”等进行品牌包装，不断提高区域性品牌知名度和竞争力。同时，坚持质量兴企、质量兴业，引导企业大力推进质量、安全、环保体系认证，加快培育一批知名企业和名牌产品。对被认定为驰名商标或省级以上名牌产品、著名商标的企业给予奖励。

全面放开搞活，搭建发展平台。坚持把培育发展面广量大的民营企业作为扩充工业经济总量的首选战略，认真落实好中央、省、市出台的一系列扶持民营经济发展的政策措施。切实保障工业用地。依托现有各类工业园区和工业集中区，加快土地资源整合，盘活存量土地，进一步配套完善工业片区基础设施。积极利用山坡地和滩涂地发展工业，拓展工业用地空间。设立工业用地成本调节基金，专项用于调节地价补贴。建设融资服务平台。进一步建立健全中小企业融资担保体系，在做大做强现有12家融资担保机构的基础上，继续在未成立担保机构的县（市）推进组建工作。鼓励商业银行创新信贷门类和服务手段，为民营企业发展营造更好的融资环境。推进行业协会建设。以行业自律、信息交流、价格协调、技能培训和营销联盟为重点，进一步完善各类行业协会的组织运作机制，充分发挥其在制定行业标准、履行行规行约和协调关系等方面的作用。加强企业人才队伍建设。落实鼓励机关干部到企业工作、挂职和鼓励人才向工业企业流动、聚集的激励政策，积极帮助企业引进高层次经营管理人才和专业技术人才。重视企业家队伍建设，今后市里每两年将举办一届优秀企业家评选活动，由市里给予表彰和奖励。着力实施“回归工程”，鼓励在外人才和企业家返乡创业。组建中小企业培训中心等培训服务机构，启动“创业者”培训工程，努力为民营企业发展提供人才支持。

3、坚持投资与消费双向拉动，促进经济持续快速增长

加快推进重点建设，保持投资较快增长。继续坚持项目带动，按照建成一批、开工一批、转化一批、储备一批、策划一批的思路，加快推进以重点建设为主导的固定资产投资。今年全市计划安排重点建设项目65个，其中重点在建项目46个，预备重点项目19个，有23个项目列入省重点项目。重点项目总投资577.5亿元，年度计划投资71.4亿元，比去年增长15.2%。重点在建项目中工业及能源项目23个，年度计划投资38.2亿元，分别占重点在建项目数和计划投资额的50%和53.5%。重点围绕工业、交通、能源、市政设施和社会事业等五个方面，做到“五个确保”：确保宁德火电厂一期2台机组、宁德电力输配电网改造建设、宁屏路和宁德市金马海堤加固工程等22个项目年内全面或基本建成投产；确保闽东宾馆迁建、闽东医院病房大楼等10个项目完成主体工程或阶段性目标；确保温福铁路宁德段、洪口水电站、东侨路网工程、宁德师专新校区、市污水处理厂和中心城市供水第二水源工程等14个项目完成年度投资计划；确保宁古路古田段、三都澳10万吨级船厂、安发国际益槐（福建）生物科技园、交溪水利枢纽工程、市垃圾焚烧处理厂、宁德医院搬迁工程、华洲水产品批发综合市场等预备重点项目开工建设；确保基本完成原定的中心城市85个城建项目年度投资任务，进一步形成新城框架，塑造城市建设新亮点，为争创卫生城市和园林城市打基础。为此，必须继续落实重点项目建设年度目标责任制和领导挂钩负责制，创新完善项目工作机制，进一步拓展项目建设资金筹集渠道，强化重点建设中的移民、征地、拆迁、补偿工作，及时协调解决好项目建设过程中遇到的困难和问题，为项目组织实施提供保障。同时，继续做好以大型临海产业、交通基础设施为主的一批重大项目前期工作，加快推进深化洽谈、立项报批和引进落地，尽快发挥重大项目的带动效应。

大力发展第三产业，着力扩大消费需求。全面实施宁德市旅游发展总体规划，着力加快资源整合、要素配套和线路对接。继续办好太姥山、白水洋旅游文化节。做好木拱古廊桥、太姥山、白水洋、三都澳、支提寺、九龙氵祭 –鲤鱼溪、中华畲族宫、临水宫等旅游精品项目包装申报工作，力争在旅游品牌建设上取得新进展，提升旅游产业规模效益。制定出台进一步促进房地产业健康发展的政策，加大对房地产市场的调控，优化住房供应结构，引导房地产开发。支持普通商品住房和经济适用房、廉租房开发，搞活房地产二级市场，鼓励市民换房买房，促进住宅消费。加强市场体系建设，针对我市商贸流通业散、弱、小的状况，加快建设汽车销售市场、IT产品、数码产品、电器产品大型专业市场和各类大型农水产品批发市场等一批商贸业项目，进一步形成教育、文化、健身、家政服务等新的消费增长点，推动消费结构升级。根据城市发展需要，加快中心城市汽车南站和配套站点建设，提高城市公交的覆盖面和密度。积极培育现代物流业，配套建设一批物流码头和物流基地，推进港口航运业特别是集装箱运输业发展。实施农村“万村千乡市场工程”，加快建立健全农村商业网点，鼓励和引导商贸服务企业深入农村集镇，发展连锁经营、农村超市、农家店等现代流通业态，拓宽消费品进入农村的渠道，挖掘农民消费潜力。落实好一系列鼓励消费的政策，清理各种抑制消费的不合理规定。深入开展整顿规范市场经济秩序工作，着力实施食品药品放心工程，坚决打击制售假冒伪劣食品、药品、农资、建材等违法犯罪行为，切实改善消费环境，保护消费者权益。

4、坚持改革与开放相互推动，促进发展活力增强

加快重点领域改革步伐。继续深化行政管理体制改革。加快政府职能转变，进一步简化行政审批，推进政务公开，促进政府工作的透明化、规范化。按照中央和省里统一部署，贯彻实施《公务员法》，改革公务员工资制度，加快推进事业单位人事和分配制度改革。深化投融资体制改革。推进投资主体多元化，拓宽融资渠道，整合资产建立市级产业发展和基础设施建设的融资平台。积极争取商业银行、银团贷款和国家政策性贷款的支持，运用引进外资、激活民资和市场化运作等方式，吸聚国际国内资本。加强政银企合作，推进金融产品创新，确保信贷投放正常合理增长。加强金融生态环境建设，维护金融稳定，促进金融与地方经济的互动双赢。推进城市管理体制改革。优化中心城市物流港口和产业港口的规划配套，合理划分蕉城区、东侨开发区的产业片区和布局。做好东侨开发区与闽东工业园区整合工作，加快推进城澳片区和漳湾片区建设，促进市本级和东侨、蕉城两区经济做大做强。创新城市管理体制，组建城市综合执法队伍，不断优化市区管理权属，充分调动各方积极性。进一步深化国有企业改革。重点推进国有内贸企业改革，加快部分国有资产改革和变现工作。稳妥推进闽东电力股份有限公司股权分置改革。加快推进财政分配体制改革，抓紧研究制定推进财政管理体制改革的具体措施。加快公共财政体系建设，调整和优化财政支出结构，促进城乡基本公共服务的均衡发展。坚持依法治税，强化收入征管，促进财政收入稳定增长。继续推进部门预算改革、国库集中支付和政府采购等制度改革，开展项目支出绩效评价试点工作。强化财政监督和审计监督，规范财经秩序，防范化解政府债务风险。今年除屏南、周宁两县财政收支少量缺口外，其余县、市、区都要实现当年财政收支平衡。

努力提高对外开放水平。加大招商引资力度。利用 “6·18”、“6·28”、 “9·8”等各种节会平台，进一步强化招商和技术对接攻势。实施多元招商，不断创新政府组织的综合性招商方式，瞄准国际国内重点地区、重点产业、重点客商，抓住大企业集团、大财团，实施上门招商、驻地招商。充分发挥涉外侨台群团和民间商会、协会的作用，积极推进以企业为主体的招商工作。推行项目链、产业链、客商链招商。建立招商人才库、合作企业库、推介项目库，加大网上招商力量。继续做好港澳台地区、海外重点地区和“长三角”、“珠三角”地区招商推介工作。做好到江西开展港口推介工作，共同推进宁武高速、宁衢铁路接通江西，加快实现三都澳成为内陆省份的出海口。加强签约项目的跟踪落实，继续实行大项目领导挂钩和专门班子负责制，切实做好服务协调工作，提高项目履约率和到资率。积极扩大外贸出口。用足用好国家和省促进出口的政策措施，鼓励引导更多企业申报进出口经营权。调整优化出口产品结构，在扩大电机、水产品等传统产品出口的同时，要在船舶业出口方面实现新突破。转变外贸增长方式，大力实施品牌兴贸、科技兴贸和市场多元化战略，积极支持企业申报技改贴息项目、开展各类质量认证和产品研发，完善检疫检验技术、检测体系，加快建立农食水产品检验室，争取设立宁德海关福安办事处，指导和帮助企业应对各类国际贸易壁垒，提高规避市场风险能力。继续加强税贸、关贸、银贸协作，不断优化通关环境，提高通关效率。加强与港澳台侨外的合作交流。按照“五缘六求”的部署，进一步巩固和扩大宁台农业、旅游、文化、劳务等领域的交流与合作，争取宁德港成为对台固定直航点。更加密切与港澳的经贸交流与合作，积极吸引港澳企业到我市开发港口、旅游等资源，发展现代服务业和物流业。继续做好以侨引侨、以侨引资、以侨引智工作，争取更多的华侨华人来宁投资兴业。

全面推进区域经济协作。继续坚持观念接轨为先导、交通接轨为基础、产业接轨为核心、企业接轨为主体的思路，以“两南”（浙南、闽东南）、“两洲”（长江三角洲、珠江三角洲）为重点，进一步推进南联北接，主动融入区域经济一体化格局，积极承接周边发达地区产业、资金、技术转移。正确处理竞争与合作的关系，立足我市海洋、生态、旅游、农副产品和水电等资源优势，进一步健全完善山海协作和区域合作机制，拓展合作领域，提升协作水平，推进传统产业互补、新兴产业共建、协同配套发展，努力把宁德建设成为周边发达地区的产业配套基地、绿色食品供应基地、休闲度假和旅游观光的后花园。

5、坚持经济与社会协调互动，促进社会全面进步

大力实施科教兴市和人才强市战略。发挥企业在技术创新中的主体作用，促进产品创新和品牌创新，提高企业核心竞争力。发挥专业技术人才在技术创新中的关键作用，对有突出贡献的优秀人才给予重奖。坚持教育优先发展，巩固“两基”成果，推进“双高普九”，重点创建一批“省级初中示范校”和“省级示范小学”。继续稳妥推进农村撤点并校，办好寄宿制中小学，加快中小学危房改造。加强中小学师资队伍建设，稳步推进基础教育课程改革。着力加快职业教育改革发展，整合市区中等职业教育资源，各县（市）重点办好1所规模大、条件好的职业学校。优化普通高中教育，做好优质高中达标晋级工作，开展校际挂钩帮扶和交流活动，促进城乡优质高中资源共享共用。加快高等教育发展步伐，积极推进宁德师专和宁德职业技术学院新校区建设，加快宁德学院筹建工作。进一步深化教育体制改革，支持民办教育，发展远程教育、学前教育和特殊教育，提高教育事业整体发展水平。高度重视人才工作，大力培养、积极引进、合理使用各类人才。充分发挥各类培训机构和职业教育资源优势，加强劳动者技术技能培训，加大力度进行专业技能人才培养和农村适用型人才培训输出工作。以项目为依托，搭建人才汇聚和创新创业的平台，大力引进急需的专业技术人才。加强人才市场建设，建立健全人才储备制度和人才柔性流动机制，完善分配制度和激励机制，用好用活现有人才，营造有利于人才成长、创业、发展的良好环境。

积极推进资源节约型、环境友好型社会建设。坚持可持续发展战略，促进经济社会发展与人口、资源、环境相协调。继续围绕争创省级、国家级计生优质服务先进县（市、区）的目标，提高人口与计划生育工作成效，稳定低生育水平。继续实施农村部分计划生育家庭奖励扶助制度和“关爱女孩行动”工程，进一步推进以小额贴息贷款为龙头的计生“三结合”工作。今年起建立“计划生育发展基金”，每年稳定在1000万元左右。坚持开发和保护相结合，集约、节约利用资源。搞好新一轮国土资源调查，抓住新一轮土地总体规划修编的契机，合理调整基本农田保护区和城市滞洪区。加强耕地保护和土地开发整理，加快滩涂围垦规划建设进度，实现当年耕地占补平衡。加大闲置土地清理力度，严格按照投资强度标准控制用地面积，提高单位土地综合利用效益。加强对港口、岸线和海域资源的保护利用，坚持规划先行，严格涉岸项目审批管理，加快清理整合不合理占用的岸线和海域资源，对开发前景良好但暂不具备开发条件的岸线，实行最严格的保护政策。强化水能资源开发与管理。严格河道采砂管理。提高石板材等重要矿产资源的集约开采水平。认真贯彻执行市长环保目标责任制，坚持预防为主、综合治理，强化从源头上防治污染和保护生态。加快垃圾、污水处理设施建设，年内完成宁德中心城市和福鼎、霞浦污水处理厂主体工程，实现福鼎、霞浦、古田垃圾无害化处理场投入使用，其余各县（市）也要加快这方面前期工作。加强重点流域水环境保护和重点区域生态治理，继续抓好海上养殖和石材业污染整治工作，致力让人民群众喝上干净的水，呼吸新鲜的空气，吃上放心的食品。加快推进殡葬改革和“青山挂白”治理。加强宁德市区公园景观绿化建设，继续推进自然保护区和国家级生态示范县、省级生态示范乡镇建设。注重水土保持，加强湿地、生物多样性和生态公益林保护，不断改善和提高生态环境质量。

加快公共文化、卫生、体育事业发展。加强城乡公共文化服务设施建设，继续完善市文化中心二期工程，启动市博物馆、图书馆、工人文化宫、闽东书画院和电影院建设项目，重点建设一批示范性的县、乡、村文化场馆，活跃社区文化、企业文化、乡村文化和校园文化。深化文化体制改革，重视文化精品创作和艺术人才培养。加大网吧治理力度。继续搞好畲族文化抢救、保护和发展工作。加强文物、文化遗产和优秀民间艺术的保护利用。做好档案、地方志工作。繁荣发展新闻出版事业。启动有线电视数字化转换工程，提高广播电视综合覆盖率。进一步健全公共卫生体系，加快突发公共卫生事件应急体系和卫生监督体系建设，做好重大传染病的预防和控制。推进城乡医疗卫生体制改革，发展民办医疗机构和社区卫生服务。积极筹备参加省第十三届运动会。广泛开展爱国卫生和全民健身运动，提高人民群众健康水平。

6、坚持民生与民安兼顾并重，促进社会和谐稳定

关心人民群众生活。认真落实国务院新一轮就业再就业政策，启动实施城乡就业保障工程，加快形成城乡一体化就业新格局。确保全年城镇新增就业2.4万人，帮助6000名下岗失业人员实现再就业。进一步扩大社会保障覆盖面，使全市城镇职工基本养老、失业、医疗、工伤和生育保险参保人数分别达到14.9万人、10.8万人、15.3万人、7.9万人和5.6万人。制定实施征地拆迁补偿办法，建立健全失地农民基本生活保障制度。完善社会救助体系，继续落实城乡居民最低生活保障制度，提高农村“五保”供养水平，对农村特困户、重点优抚对象和革命“五老”人员实施医疗救助，农村社会保障综合覆盖率20%以上。大力发展福利和慈善事业，扶持民办福利机构发展，逐步推广“慈善超市”捐助模式。加强防灾减灾体系建设，建立完善各类突发重大灾害应急预案和紧急救助体系。加快推进宁德城区防洪防潮工程和赛江防洪工程等一批重点城防工程建设。认真做好灾区群众生活救济、生产恢复和灾后重建工作。进一步深化老区优先行动，改善库区移民生产生活条件，支持少数民族地区、贫困山区、边远地区和岛屿突出部地区加快发展。继续实施“造福工程”和地质灾害点、滑坡险情村搬迁，确保全年搬迁6000人以上。

深化“平安宁德”建设。继续实施社区（村居）“11221”工程，建立健全覆盖全市城乡的社会治安防控体系。落实社会治安综合治理各项措施，继续开展对“黄赌毒”、“六合彩”等突出问题的专项治理，严厉打击各种违法犯罪活动和地下宗教势力、境外敌对势力的渗透破坏活动，坚决取缔各种邪教组织，切实维护国家安全和社会政治稳定。启动“五五”普法工程，继续开展基层“百千万”依法普法教育活动。加强群众来信来访和人民调解工作，依法及时合理地处理群众反映的问题，妥善处置各类群体性事件，引导群众以理性合法方式表达诉求，解决矛盾纠纷。加强安全生产基础工作，严格安全生产责任制和责任追究制，防止重特大事故发生，创造更加和谐稳定的社会环境。

营造民主团结氛围。扎实推进依法治市，加强民主法制建设，自觉接受人大及其常委会的法律监督、工作监督和人民政协的民主监督，认真办理人大代表建议和政协提案，广泛听取民主党派、工商联、无党派人士及各人民团体意见。推进基层民主政治建设，加强村民自治和社区民主建设，切实保障广大人民群众的民主权利，促进和谐社区、和谐村镇建设。深入开展群众性精神文明建设活动，加强公民道德建设，强化未成年人思想道德建设和青少年思想政治教育，推进“诚信宁德”建设，在全社会营造爱我宁德、建设宁德的良好风气。做好双拥工作，落实各项优抚政策。做好复员、转业军人安置工作，妥善协调解决随军家属户口和子女入学问题。强化国防教育，加大对人民武装和国防后备力量建设的支持力度，加强民兵预备役部队和人防工作。积极支持工会、共青团、妇联等群众团体依照法律和章程独立自主开展工作，更好发挥其联系社会的桥梁和纽带作用。认真贯彻党的民族、宗教政策，依法管理宗教事务，促进民族共同团结奋斗、共同繁荣发展。关心支持和重视做好民政、人事、编制、审计、统计、地震、水文、气象、老干、老龄、残疾人事业及关心下一代等工作，充分调动各方面的积极性、主动性和创造性，形成全市上下团结奋斗、干事创业的良好局面。

三、突出服务，努力建设高效清明的人民政府

各位代表，从2006年开始，我们迈入了不同寻常的“十一五”时期。历史的重责，人民的重托，发展的重任，要求我们必须进一步改进作风，转变职能，创新服务，提高效率，努力把各级政府建设成为高效清明的人民政府，为加快发展创造更为宽松优良的环境。

我们要始终做到开明务实、创新有为。主动把宁德的发展放在全国、全省的大局中去谋划、去把握、去推动，加强对经济社会发展中重大问题的研究，自觉将中央的方针政策和省委、省政府的重大决策部署与工作实际结合起来，向改革要办法，因地制宜找准加快发展的切入点；靠创新找出路，与时俱进推出促进发展的新举措；以实干求实效，积极有为打开推动发展的突破口。当前，特别要盯住影响加快发展的征地拆迁难、项目落地难、融资难、环境优化难等难题，善于从重点部位、重点环节入手，从矛盾的焦点入手，从关键性、全局性、根本性问题入手，一个一个地研究探索，一个一个地突破解决，把我们的事业一步一步地推向前进。

我们要始终做到执政为民、优质服务。进一步强化“民本”意识、“公仆”观念，自觉把最广大人民群众的利益作为制定政策、开展工作的出发点和落脚点。坚持亲民作风。经常深入基层、深入群众、深入生产第一线，倾听民声，尊重民意，了解民情，关心民众。办好惠民实事。认真办好为民办实事项目，解决好与人民群众生产生活密切相关的热点难点问题。不折不扣地落实好中央、省、市出台的农村税费改革、新型合作医疗、九年义务教育、最低生活保障等各项惠民政策，切实维护和保障群众的合法权益。创新便民措施。按照“规范程序、简化环节、明确权限、承诺服务、限时办结”的要求，继续深入开展“行政提速、服务发展”民主评议活动，大力加强市投资服务中心等政府机关直接面对群众和企业的“第一道门槛”建设，完善“一站式”办公、“一条龙”服务，实行项目委托代理制和限时办结制，解决好项目审批“多头跑”问题，切实方便群众、方便企业、方便投资者。

我们要始终做到高效运作、真抓实干。做好政府工作，科学决策、民主决策是前提，运作有效、落实有力是保障。坚持领导带头抓落实。各级领导干部要各负其责，深入一线，靠前指挥，跟踪落实。特别是对重大的招商引资项目、基础设施项目，更要主动协调，现场办公，一件一件抓落实，一环一环严督查，一项一项求突破出成效。完善责任机制求落实。进一步建立健全执行和督查落实机制，着力从组织、流程、人员、责任等方面，构建有效的执行体系，建立健全督查到岗、考核到人的工作推进机制；加强行政效能建设，严格执行工作责任制和责任追究制，确保各项决策不折不扣地得到执行，落到实处。抓好具体细节促落实。重具体、重细节，历来是成功之道。要细化目标，深化举措，量化考核，对每一项重点工作，都要逐个设计可以量化、可以操作的载体，制定具体的工作计划和考核办法，从具体事做起，从细节处着手，以具体之行见责任，在细节之中见成效。

我们要始终做到依法行政、廉洁从政。把依法行政作为政府工作的基本准则，切实增强各级政府领导干部和公务员的法制观念。以贯彻实施《公务员法》为契机，切实加强公务员队伍管理，努力建设一支高素质的公务员队伍。坚持依法决策，依法办事，严格依照法定权限和程序行使权力、履行职责、接受监督，做到既不失职，又不越权。不断深化行政执法体制改革，从制度上解决多头执法、多层执法和执法真空等问题。认真贯彻落实《关于建立健全教育、制度、监督并重的惩治和预防腐败体系的实施条例》，建立有效的权力制约机制，加强对权力运行的制约和监督。加大对违法违纪案件的查办力度，坚决惩处腐败分子。坚持纠建并举、综合治理，加强政风行风建设，坚决纠正损害群众利益的不正之风。继续深化政务公开工作，强化行政监察和审计监督，努力从源头上预防和治理腐败，树立起良好的清廉政府形象。

各位代表，“十一五”时期，是宁德实现新跨越、再上新台阶的关键时期。2006年是我们站在新起点、肩负新使命的开局之年。历史召唤我们，时代激励我们。我们要坚持以科学发展观为指导，在市委的坚强领导下，盯紧发展，抢抓机遇，只争朝夕，开拓奋进，为加快推进海峡西岸经济区东北翼中心城市建设而努力奋斗！
